# Ionic amplifying circuits inspired by electronics and biology

**DOI:** 10.1038/s41467-020-15398-3

**Published:** 2020-03-26

**Authors:** Rachel A. Lucas, Chih-Yuan Lin, Lane A. Baker, Zuzanna S. Siwy

**Affiliations:** 1 Department of Physics and Astronomy, University of California, 4129 Frederick Reines Hall, Irvine, CA 92697 USA; 2 Department of Chemistry, Indiana University, 800 E. Kirkwood Avenue, Bloomington, IN 47405 USA; 3 Department of Chemistry, University of California, Irvine, CA 92697 USA; 4 Department of Biomedical Engineering, University of California, Irvine, CA 92697 USA

**Keywords:** Electronic devices, Electronic devices

## Abstract

Integrated circuits are present in all electronic devices, and enable signal amplification, modulation, and relay. Nature uses another type of circuits composed of channels in a cell membrane, which regulate and amplify transport of ions, not electrons and holes as is done in electronic systems. Here we show an abiotic ionic circuit that is inspired by concepts from electronics and biology. The circuit amplifies small ionic signals into ionic outputs, and its operation mimics the electronic Darlington amplifier composed of transistors. The individual transistors are pores equipped with three terminals including a gate that is able to enrich or deplete ions in the pore. The circuits we report function at gate voltages < 1 V, respond to sub-nA gate currents, and offer ion current amplification with a gain up to ~300. Ionic amplifiers are a logical step toward improving chemical and biochemical sensing, separations and amplification, among others.

## Abstract (web summary)

Though ionic circuits have been reported, previous technologies did not allow ionic output or operate at significant voltages. Here, the authors, inspired by concepts from electronics and biology, show an abiotic ionic circuit that functions at voltages below 1 V and amplifies sub-nA ion currents.

## Introduction

Integrated circuits (ICs) revolutionized our lives, and are ubiquitous in virtually all electronic devices including cell phones, computers, and pacemakers^1,2^. ICs consist of electronic components such as diodes and transistors, and allow for signal manipulation and amplification. On the other hand, the physiological processes of living organisms rely on another type of circuit, which is entirely ionic and functions in an aqueous environment^3,4^. The key players in physiological processes are biological channels in a cell membrane that facilitate exchange of ions and molecules, for instance between the intracellular and extracellular spaces in cells and tissues. This transmembrane ionic transport is often the first step in a biological amplification process, which enables sensing external stimuli including light, sound, and odor. In the signal transduction of sound, for example, hair cells of the cochlea mechanically transduce sound waves into ion currents by opening cochlear ion channels to ionic transport; open channels allow millions of ions to pass through per second, which leads to signal generation (in the form of a change in transmembrane potential), and is ultimately detected and processed by the brain^5^.

Examples of man-made ionic circuits have already been reported. One of the first ionic circuits was composed of four biological α-hemolysin channels, where individual channels had been chemically modified so that they functioned as ionic diodes^6^. Connecting four such channels into a circuit known from electronics as a bridge rectifier enabled changing alternating current into direct current. In another example, ionic diodes based on rectifying nanopores and microchannels were connected into logic gates^7–9^. Logic gates composed of microfluidic ionic transistors have also been designed^10^. Among ionic circuits reported, those that utilize organic electrochemical transistors (OECTs) have attracted significant interest from both fundamental and applied perspectives due to the high amplifications and speeds of operation they offer^11,12^. In OECTs injection of ions from solution leads to modulation of conductivity of a semiconductor or conducting polymer. OECTs offer large amplifications, because small ionic signals are amplified into a large electronic current^13^. Organic electrochemical transistors have found numerous applications, which include printed integrated circuits, biological interfaces, and neuromorphic devices.

OECTs enable natural communication between ionic devices—so-called iontronics—and electronics but due to electronic output, they do not offer amplification in an ionic manner. It would be of great interest however to design circuits that amplify small ionic currents such that the output is ionic as well. Such a system would be fundamentally different in operation from OECT based devices and may find utility when the chemical identity of the output is important, and when that output has to be delivered to a specific part of the circuit. In principle ionic amplification is observed in the classic biological example of spatially and chemically complex ion transport that occurs in the synaptic cleft, where signaling molecules, driven by a propagating action potential, are released from the presynaptic terminal, diffuse across the synaptic cleft, and are taken up by receptors on the postsynaptic terminal^14^. This process—selective release (e.g., from vesicle fusion at the presynaptic terminal), confined transport of ions in space (in the synaptic cleft), and selective uptake (e.g., a specific ion channel receptor in the postsynaptic cleft), provides an ultimate example of what can be realized with gated and selective transport of ions. While abiotic systems cannot presently approach this level of sophistication, we can provide simplified, layered designs to construct synaptic cleft inspired devices. Chemical selectivity at the level of counter ion charge (positive/negative) is achieved in nanopores with excess surface charge or in thin polymer films^15^. The nanopore itself provides a confined transport space, which enhances the effects of space charge from counter ions. We see this admittedly crude initial step as a simple example of how models of controlled ion/molecule transport, an approach with widespread utility in biological systems, can be combined with principles from electronic devices to realize ionic amplification or switching of chemical systems.

Ionic amplifiers with ionic output could also enable local delivery of molecules used, for example, in controlling local polymerization^16^, as suggested in an earlier work^10^; more generally, delivery of different molecules at different parts of ionic circuits would lead to spatially and temporally complex patterns of chemical reactions, and emergent phenomena^6,17–19^. In addition, if ionic transistors are based on channels in a membrane, they could be used in setups that mimic biological systems and enhance transmembrane ionic flow^20–26^.

The first realization of the concept of ionic amplification was achieved in individual microfluidic ionic transistors. In an ionic bipolar transistor composed of cation and anion selective polymers, an ionic base current was amplified to an ionic output with a gain of 10 using an emitter-base voltage of 4 V^21^. An ionic transistor was also realized in a microfluidic system with an anion exchange membrane, where voltage applied across the membrane induced concentration polarization in the output channel that either enhanced or stopped ionic flow^27^. This transistor offered a gain of 45 but due to a macroscopic length of the device, voltages as high as 20 V had to be applied across the device.

Here, we show an ionic and tunable amplifier that functions at voltages below 1 V, making it compatible with biological signals. The amplifier uses inputs and outputs that are ionic. In contrast to the previous ionic transistors based on microfluidic systems^21,27^, devices proposed here are based on nanopores embedded in films. Tunability of amplification comes from the modular character of the circuits. The amplifying circuits we present were inspired by both electronic amplifiers and biological signaling pathways. As electronic amplifiers often contain two or more bipolar transistors, we first prepare individual ionic transistors, which are then connected in a multicomponent circuit known from electronics as the Darlington amplifier. Darlington amplifiers offer greater amplification of currents than individual devices^1^. The amplifying ionic circuits that we propose are applicable to sensing ion currents down to 100 pA in 100 mM KCl at pH values relevant to physiological conditions.

## Results

### Fabrication of ionic bipolar junctions

**Fig. 1:**
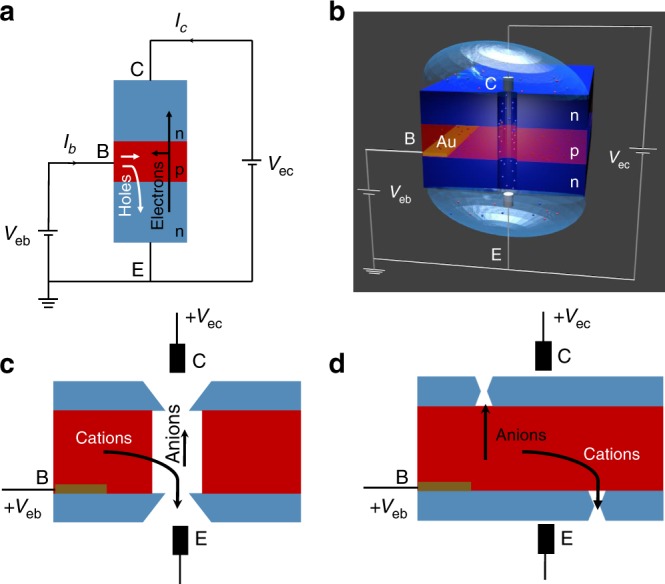
Design of an ionic bipolar junction. **a** Scheme of a solid-state npn bipolar junction transistor in the active mode; C, E, and B indicate collector, emitter and base, respectively. V_eb_ and V_ec_ denote voltage between emitter and base, and emitter and collector, respectively. I_c_ is the collector current, and I_b_ is the input current. **b** Scheme of an ionic equivalent of an npn transistor with voltages, V_eb_, V_ec_, as applied in the experiments. V_eb_ is applied through the Au gate electrode shown in orange. Blue regions of the device correspond to a zone with positive surface charges that lead to accumulation of anions (marked as blue dots); the red region is negatively charged and filled with cations (marked as red dots). A zone filled with anions is an ionic equivalent of an n doped semiconductor; a zone filled with cations is an ionic equivalent of a p doped semiconductor. **c** Arrangement of two 40 nm thick silicon nitride chips (shown in blue) and the Nafion film (red) used in the ionic transistor. A pore through the silicon nitride and Nafion sandwich was drilled by focused ion beam. Silicon nitride was modified chemically to render the surface positively charged. Nafion is negatively charged thus expected to be filled with cations. **d** Device scheme for the transistors with smaller pore size. This transistor was assembled using two silicon nitride chips with nanopores drilled prior to the device assembly. A Nafion layer is present between the two chips, similar to the arrangement shown in **c**.

The ionic bipolar transistor we designed is a nanopore, which consists of two diode junctions connected via the base region^28,29^. We report here the preparation of an ionic equivalent of a semiconductor npn device (Fig. 1a), in which two zones with positive surface charges are connected to a middle negatively charged zone (Fig. 1b). In low salt concentrations, the zones with positive charges will have enriched concentration of anions, creating an ionic equivalent of an ‘n’ doped semiconductor; the middle zone will be filled with positive charges, creating an ionic mimic of a ‘p’ doped semiconductor^15^. The system contains three electrode terminals to control the bias of the two diode junctions (Fig. 1b)^10,21^. Similar to electronic transistors, the external voltage signals can be tuned to allow ionic flow through two, one, or none of the junctions. If both junctions allow ionic transport, the device is in an on-state, called saturation mode. If only one junction allows current flow, the mode of operation is called active mode, and the transistor can amplify the signal. Finally, when both junctions form depletion zones the device is in the off-state, or cutoff mode.

The schemes of the ionic npn transistors we designed are shown in Fig. 1c, d. The fabrication process we developed utilizes two 40 nm thick silicon nitride films on a silicon support with a Nafion layer sandwiched between them. The Nafion layer was spin-coated and its thickness was estimated to be ~1 μm (Supplementary Fig. 1). The device has three terminals called the emitter (E), which in our case is the ground, the collector (C), which provides the working voltage across the device, V_ec_, and the base (B), which provides voltage, V_eb_, to the gate electrode in the center of the device (Fig. 1b). The gate electrode is a 10 nm layer of gold that was sputter-coated onto one of the silicon nitride surfaces.

Two fabrication routes were developed to create a well-defined ionic path in the system. In the first approach, a Focused Ion Beam (FIB) was used to drill a pore through the entire silicon nitride/Nafion/silicon nitride sandwich structure (Fig. 1c)^30^. Due to the micrometer-scale thickness of the assembled sandwich structure, the pore diameter drilled was at least 200 nm to ensure the pore extended through both silicon nitride films and the Nafion. Eight devices with pore openings between 200 nm and 600 nm were prepared. Due to the damaging effect of ion beam on polymers, it is expected that the zone with Nafion will have an opening that is larger than the pore diameter in silicon nitride. We estimated the difference in the opening would not exceed the factor of three (Supplementary Fig. 2). The device fabrication was completed by rendering the silicon nitride external surfaces and the pore walls positively charged. A silanization reaction with amine terminated silanes was performed as reported before^31^; this reaction targeted only silanol groups present on silicon nitride thus it was not expected to modify Nafion. In the complete ionic npn transistor, the film of Nafion plays the role of base and cation source^32^. When positive voltage is applied to Nafion, cations will be injected into the pore enhancing the collector current; with negative gate voltage, cations will be attracted to the area of Nafion, which will lead to reduction of the transmembrane signal.

To prepare devices with smaller opening diameter, we first used FIB to drill single nanopores in two separate silicon nitride chips, and subsequently subjected them to the silanization reaction to introduce amines at the surface. In this case, drilling only through 40 nm thick films was performed, thus smaller nanopores down to ~100 nm were obtained. Both chips were subsequently spin-coated with Nafion and assembled into a device under a light microscope (Fig. 1d, Supplementary Fig. 3). Note that in this case, the nanopores in the two silicon nitride films were not aligned; however, due to low resistance of the Nafion film and its large area we expect ions can be injected to the pore region over many voltage cycles.

### Performance of individual ionic bipolar junctions

**Fig. 2:**
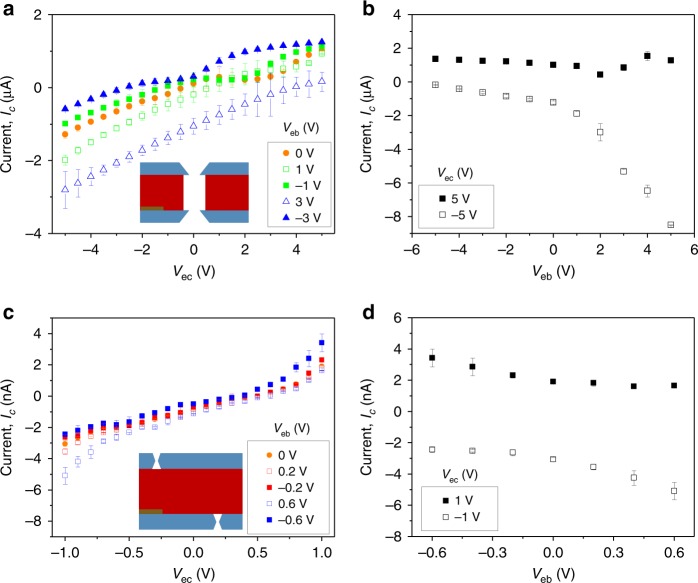
Current-voltage curves of individual bipolar junctions at various base voltages. **a**, **b** Recordings for a transistor with an opening diameter of 600 nm in 10 mM KCl; (**c**, **d**) recordings for a device with an opening diameter of 140 nm in 100 mM KCl. **b**, **d** were created based on current-voltage curves in **a** and **c** and show I_c_ at chosen V_ec_ for different gate inputs. Error bars were calculated as standard deviation from an average of at least two independent voltage scans.

The ionic npn transistors were characterized in a conductivity cell whose two chambers were filled with the same concentration of KCl at pH 8. Example sets of characteristic curves for two devices with a diameter of 600 nm and 140 nm are shown in Fig. 2 for 10 mM and 100 mM KCl, respectively. Successful preparation of the devices and their chemical modification is evidenced by recording I–V curves between the base and emitter, with the collector terminal disconnected (Supplementary Fig. 4)^21^. With three electrodes connected as shown in Fig. 1b, the devices exhibit ion currents with magnitudes that can be tuned by the base input voltage, V_eb_. Analysis of these recordings is facilitated by plotting the current value, I_c_, at a given collector voltage, V_ec_, for each base voltage input (Fig. 2b, d). The first difference between these two devices is the range of base voltages to which they responded. The 600 nm device was able to modulate ion current only for gate voltages larger than or equal to 1 V, and was functional at V_ec_ > 1 V. The 140 nm transistor responded to gate voltages as low as 0.2 V with V_ec_ = 1 V. These two transistors also functioned at different salt concentrations—the smaller transistor was probed in 100 mM KCl, while the 600 nm device responded to gate voltage in ten-fold lower concentration of 10 mM KCl. Recordings and modeling of the 600 nm transistor in 100 mM KCl are included in Supplementary Figs. 5–8, and indicate that the transport properties of the device were modulated only by V_eb_ = ±5 V.

**Fig. 3:**
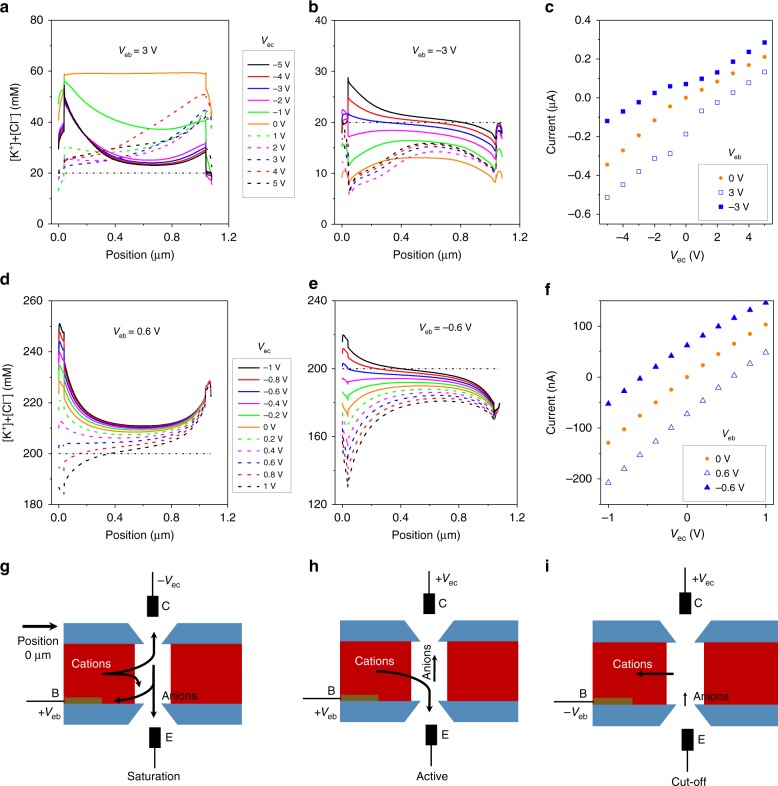
Numerical modeling of ionic concentrations in a bipolar junction transistor. **a**–**c** Analysis of a device with a 500 nm in diameter pore in 10 mM KCl, pH 8, schematically shown in Fig. 1c. Concentration profiles along the pore axis for **a** V_eb_ = 3 V, and **b** V_eb_ = −3 V, and V_ec_ varied as shown in the legend. **c** Current-voltage curves predicted from the model for V_eb_ = ±3 V. **d**–**f** Analysis of a device with 150 nm in diameter pores in 100 mM KCl, pH 8. Scheme of the device is shown in Fig. 1d. Concentration profiles along the pore axis for **d** V_eb_ = 0.6 V and **e** V_eb_ = −0.6 V, and V_ec_ varied as shown in the legend. **f** Current-voltage curves predicted from the model for V_eb_ = ±0.6 V. All concentrations shown are cross-section averaged. A dashed-dotted line in **a**, **b**, **d**, and **e** indicates bulk concentration of ions. The collector is placed at the position 0 μm. Note that the pores in the two transistors modeled were assumed to be aligned (Supplementary Fig. 5) to assure axial symmetry of the system. **g**–**i** Schemes of saturation (**g**), active (**h**), and cutoff (**i**) modes of operation of an ionic transistor.

Figure 2 shows that the characteristics of the two npn devices depend not only on the polarity of the gate voltage but also polarity of V_ec_. Analysis of the experimental recordings is facilitated by modeling of ionic concentrations and ion current performed by numerically solving coupled Poisson-Nernst-Planck and Stokes-Brinkman equations (Fig. 3, Supplementary Figs. 5–10, Supplementary Notes 1 and 2). Two different geometrical models representing structure of the two devices were developed with the Nafion region considered as a zone with volume charge density (Fig. 1c, d, Supplementary Fig. 5)^32^. The pore diameter in the larger device was assumed 500 nm; the model of the smaller device (Fig. 1d, Supplementary Fig. 5b) contained nanopores with 150 nm in diameter opening. The bulk concentrations used in the modeling in Fig. 3 were the same as bulk concentrations in experiments shown in Fig. 2. With V_ec_ < 0, the positively biased gate is at the highest potential so that the two junctions are in the on-state with potassium flowing from the gate region towards collector and emitter. This voltage configuration produced the highest currents in both experiments and the models for the two devices, and represents the saturation mode (Fig. 3g). In this mode of operation, both diode junctions of the transistors are in the on-state, ionic concentrations in the devices are higher than the bulk and increase with the increase of the gate signal. The case of V_ec_ > 0 and V_eb_ > 0 is expected to correspond to the active mode of the bipolar junctions in which one of the diode junctions is in the off-state (Fig. 3h). The ionic concentrations confirm the predictions and clearly reveal the region with ionic depletion that corresponds to the off-state of the diode junction located at a position of ~50 nm (Fig. 3a, d). When V_eb_ and V_ec_ are negative, the devices can work in the reverse active mode, which is also expected to provide amplification; in this voltage configuration, the emitter-base diode junction is a depletion zone (Fig. 3b, e). In these two active voltage regimes, the gate signal can modulate ionic concentrations in the device to provide ion current amplification. Finally for negative base voltages and positive transmembrane potential, the transistor should be in its cutoff mode with negligible values of the collector current (Fig. 3i). However, experiments and simulations demonstrated that the ionic bipolar junctions we designed would conduct finite current in this mode as well, which we believe stems from the nanoscale thickness of the silicon nitride films. As predicted before, when a short zone with a surface charge of one polarity (positive charges on silicon nitride in our case) is connected to a thicker ion selective region of the opposite charge polarity (Nafion), the ability of this diode junction to create an off-state is maintained only for small voltage magnitudes^33^. In addition, the pore opening of at least 100 nm prevents formation of a fully formed depletion zone so that the finite ionic concentrations in all voltage arrangements lead to ion current.

Our experiments and modeling confirm that ionic transistors based on pores as large as a few hundred nm in diameter were functional and responsive to V_eb_, as evidenced by voltage-modulated ionic concentrations in the pores, and were consistent with earlier modeling of ionic junctions^33^. As an example, for the 500 nm in diameter pore in the saturation mode at V_eb_ = 3 V and negative V_ec_ (Fig. 3a), ionic concentrations were locally enhanced even three-fold compared to the bulk. The depletion zone shown in Fig. 3b for negative V_ec_ contained ionic concentrations that were half the bulk value. Supplementary Fig. 8 shows modeling performed for the same ionic transistor based on 500 nm pores in 100 mM KCl. As expected, modulation of ionic concentrations was weaker when the device was in contact with the more concentrated salt; the depletion zone for negative base voltage and negative V_ec_ was still observed, however the ions were depleted only by ~15% compared to bulk (Supplementary Fig. 8d). The transistor was not able to display a saturation mode, so that ionic concentrations at V_eb_ = 3 V and negative V_ec_, stayed at values that were similar to the bulk concentration. (Supplementary Fig. 8c).

We looked in more detail at the active mode of the ionic bipolar junction. Due to the presence of high ionic concentrations in the zone of the pore in contact with Nafion (the gate), the collector, emitter, and gate zones are ionically connected. Consequently, the collector current, I_c_, is also affected by the relative magnitude of the voltages V_eb_ and V_ec_. When V_eb_ is low, the gate signal will amplify the ionic flow between emitter and collector, as is the case in an electronic transistor. As V_eb_ increases, ionic connectivity between the transistor zones increases the ionic flow between emitter and gate leading to diminished collector signal, I_c_, and diminished ionic amplification. Consequently, the ionic bipolar junction is expected to be most powerful in amplifying small gate signals. The ionic connectivity of the device components might also contribute to the finite current in the voltage arrangement corresponding to the off-state, since V_eb_ and V_ec_ are of opposite polarity enhancing the voltage drop over the diode junction.

The saturation mode of the 500 nm device modeled in 10 mM KCl was also analyzed in more detail. We noticed that at V_eb_ = 3 V, and V_ec_ range between 0 V and −3 V, ionic concentrations decreased as V_ec_ became more negative. As an example, ionic concentrations at V_ec_ = −1 V were higher than at V_ec_ = −3 V. We believe this effect is caused by the high magnitudes of both V_eb_ and V_ec_ applied to this device (Supplementary Figs. 9 and 10). As V_ec_ becomes more negative, the voltage difference between V_eb_ and V_ec_ increases as well, demanding more cations. Consequently, in the pore region adjacent to the Nafion, a zone with lower ionic concentrations is formed, especially pronounced at positions between 0.4–0.6 μm (Fig. 3a). Note however that at the voltage configuration corresponding to the saturation mode of the device, ionic concentrations are enhanced compared to the bulk at all positions along the axis for all voltage magnitudes examined.

Modeling of ionic concentrations in the 150 nm device revealed that this device could also demonstrate all four operation modes of a bipolar junction. The predicted magnitude of I_c_ was however significantly higher than values observed experimentally (Figs. 2c, 3f); recordings for another device are shown in Supplementary Fig. 11. To find the origin of the discrepancy we first took into account the possibility that the two chips with nanopores were not aligned, as shown in Fig. 1d. A 3-dimensional numerical model was built to calculate ion current where the two nanopores were off-set by a distance of 500 and 750 nm. The modeling revealed that the predicted current was independent of the respective positions of the two nanopores (Supplementary Fig. 12). We also considered consequences of the presence of a highly conductive Nafion membrane in the distributions of electric field in the ionic transistor. In the transistors with smaller pores (Fig. 1d), the ion selective membrane is placed between the two nanopores, and consequently it separates fluxes of cations and anions sourced on the opposite sides of the membrane^34^. As explained in previous publications, the presence of such a membrane induces concentration polarization and formation of a dynamic depletion zone with locally inhomogeneous ionic concentration^35^. The depletion zone creates an additional resistance in the system that could be responsible for the diminished experimental magnitude of I_c_. Including the dynamic and spatially inhomogeneous depletion zone into the modeling would require incorporating possible destabilization of the depletion zone by local vortices, which is beyond the scope of this work^36^.

### Ion current amplification with ionic bipolar junctions

**Fig. 4:**
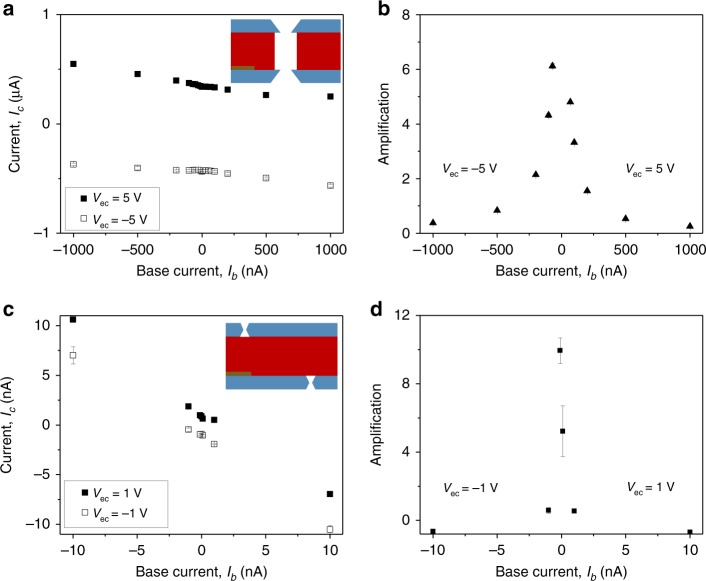
Amplification performance of ionic bipolar junctions. **a**, **b** Measurements for an ionic transistor with an opening diameter of 540 nm in 1 mM KCl. **a** Current, I_c_, at a collector voltage of either V_ec_ = −5 V or V_ec_ = 5 V as a function of base current, I_b_. **b** Current gain calculated as a function of base current. Values calculated for V_ec_ = −5 V at negative base currents, and V_ec_ = 5 V at positive base currents correspond to the active mode of the device. **c**, **d** Measurements for an ionic transistor with an opening of 140 nm in 100 mM KCl. **c** Current, I_c_, at a collector voltages of −1 V and +1 V as a function of base current together with current gain shown in **d** calculated at V_ec_ = ±1 V. Error bars were calculated as standard deviation from an average of values in at least two voltage scans.

To facilitate characterization of the transistors by amplification, the base input signal was changed from voltage, V_eb_, to current, I_b_. Figure 4a, c shows dependence of a collector current, I_c_, on I_b_ for two devices, with an opening diameter of 540 nm in 1 mM KCl, and the 140 nm device in 100 mM KCl; the 140 nm device is the same as shown in Fig. 2c. A lower ionic strength had to be used for the larger device to observe the amplification effect. Figure 4b, d shows current gain, calculated as $\beta = \frac{{I_{\mathrm{c}}}}{{I_{\mathrm{b}}}}$ where I_c_ is the output current through the device and I_b_ is input current into the base (Fig. 1). All calculations were performed in the active mode of the devices. Amplification of the 540 nm in diameter transistor was determined at V_ec_ = +5 V and positive I_b_ as well as for V_ec_ = −5 V and negative I_b_, correspondingly. The 140 nm in diameter transistor provided amplification at V_ec_ = 1 V and V_ec_ = −1 V. The gain values reported were calculated only for the magnitudes of I_b_ for which the difference (*I*_c_(*I*_b_) − *I*_c_(*I*_b_ = 0)) was larger than the average error of measuring I_c_. The highest gain value for the 540 nm device was ~6, and the smallest current detected and amplified was 70 nA. The 140 nm transistor provided similar amplification values however the detected base currents were 70-fold smaller, ~1 nA, in a hundred times higher ionic strength solution. Note however that the ionic transistors based on narrower pores offered significantly higher amplifications when V_ec_ was increased from V_ec_ = ±1 V to a value of V_ec_ = ±3 V. Figure S13 shows results for a transistor containing 155 nm in diameter pores that at V_ec_ = ±3 V provided amplification of 1 nA currents with a gain of 300.

### Ion current amplification with a Darlington pair

**Fig. 5:**
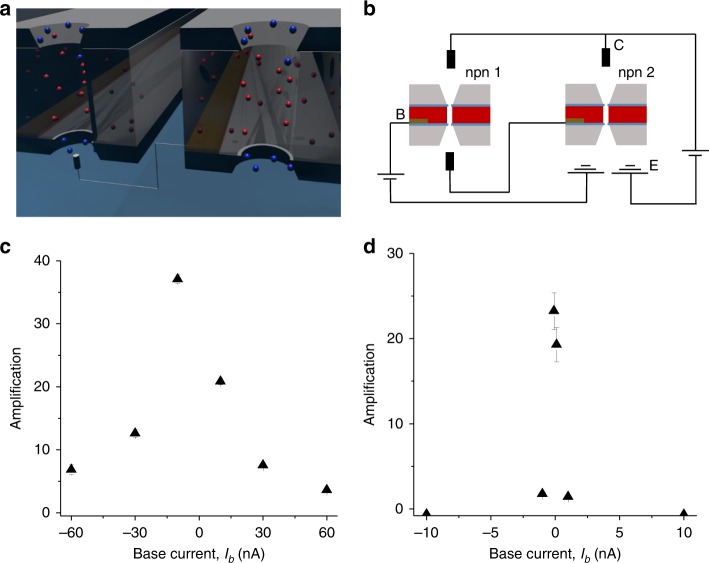
Amplification performance of an ionic Darlington circuit. **a**, **b** Darlington amplifier circuit scheme and setup, respectively. **c** Current gain as a function of base current, I_b_, for two bipolar transistors connected in a Darlington amplifier circuit in 1 mM KCl. Devices had pore sizes of 540 nm and 520 nm. **d** Current gain for a Darlington circuit composed of two bipolar transistors that contained pores with diameter of 140 nm and 150 nm; measurements were taken in 100 mM KCl. Error bars were calculated as standard deviation from an average of values in at least two voltage scans.

It is known from electronics that current can be amplified to a significantly higher degree through the use of a Darlington amplifier circuit setup^1^. A Darlington amplifier consists of two transistors connected such that the output of the first transistor becomes the input of the second one (Fig. 5a, b). This method can provide amplifications in solid-state transistors as large as the gains of the individual devices multiplied plus the gain of each device individually.

Performance of two pairs of ionic transistors connected in a Darlington circuit is shown in Fig. 5c, d. The device studied in Fig. 4a, b and another transistor based on a 520 nm in diameter pore (Supplementary Fig. 14a) were used in the first circuit. The circuit offered amplification as high as 40 (Fig. 5c), thus over six times higher than the amplification of an individual transistor shown in Fig. 4b. An important advantage of the Darlington circuit we built is the ability to probe lower I_b_ current magnitudes compared to individual devices. Consequently, the same base voltage range produces larger amplifications at lower input currents for the Darlington setup than for an individual npn transistor; note the difference in I_b_ input in Figs. 4a, b and 5c.

Figure 5 also shows performance of another pair of ionic transistors based on two nanopores with an opening of ~150 nm (Fig. 4c, Supplementary Fig. 15a). This Darlington circuit amplified the ion current in 100 mM KCl and offered a gain of 25, which is three-fold higher than amplification of one of the transistors when probed alone (Fig. 4d). The smallest current detected by the circuit was 0.1 nA thus 10-fold lower than currents detected by an individual transistor shown in Fig. 4c.

The two individual npn bipolar junctions used in the Darlington circuits were characterized with different gains, and we realized that their position in the circuit mattered for the overall amplification. Figure 5c, d corresponds to the cases when a device with smaller amplification provided the first amplification step; as mentioned above, the Darlington circuits provided gains as high as 40 and 25, respectively. The two cases with the position of individual bipolar junctions switched are shown in Supplementary Figs. 14b and 15b, and indicate that when the better performing transistor is placed in the circuit first, the overall performance is lower (gains of 7 and 15). The origin of this effect will be investigated in the future.

We tested 16 individual ionic transistors, and the most sensitive Darlington setup we prepared with these devices was able to amplify currents as small as 100 pA, Fig. 5d. Future work will tune device pore size and base thickness so that smaller currents will be detectable.

## Discussion

In conclusion we demonstrated preparation of ionic circuits that were inspired by systems in biology and electronics. The ability to build individual ionic transistors and combine them into easily reconfigurable amplifying units will enable preparation of ionic equivalents of electronic amplifiers including an operational amplifier. Increase of the sensitivity of the individual transistors and circuits to levels as low as fA will require further miniaturization of the system, especially the gate region, and increasing ionic selectivity of all zones. The ionic circuits described in the manuscript present an example design how to achieve ionic amplification, and can facilitate preparation of artificial biocircuits such as neuronal signaling. For amplification systems reported here, ionic transistors are nominally charge selective, meaning they respond only to the net charge of the ion in the electrolyte. In contrast, biological channels can differentiate between ions of the same charge, such as potassium and sodium^3^. One can envision, however, that the ability to impart chemical selectivity at specific locations along the nanopore could also be incorporated into this platform, as recently shown through crown-ether functionalization of nanopores^37^, and crown-ether channels^38–41^. Such ion selective channels would enable a selective response of the amplifying circuits to specific ions. It is also possible to attach receptors such as specific DNA sequences or aptamers^42–44^ to sequester chosen ions, or enhance or diminish the local double layer, and in turn ion concentrations inside of the nanopore^45,46^. In such systems, the changes to the local electrochemical potential might ultimately be subtle, which makes transistor designs especially appealing in terms of providing a possible route to signal amplification. Introducing ion selectivity into ionic amplifying systems will be pursued by us in the future to bring ionic, experimental models of neuron signaling closer to reality.

## Methods

### Preparation of gate electrode

Fabrication of an ionic transistor began with a single 40 nm thick silicon nitride chip. Silicon nitride chips were home-made with a window size of 50 × 50 μm. In the first step, a mask was placed over ~3/4 of the chip before sputter coating ~10 nm of Au onto the surface of the chip to create a gold electrode. The mask was then removed and a piece of Cu tape was used to establish the connection to the Au electrode.

### Addition of Nafion

A commercially available solution of Nafion® (1100 EW, 20 wt%, ethanol based, Fuel Cell Store) was used to prepare an 8 wt% solution of Nafion in dimethyformamide (DMF) through addition of small amounts of DMF, and boiling off the alcohol. The DMF-Nafion solution was then spin-coated onto the chip bearing the Au electrode at 2500 rpm for a total of 80 s. The chip was then heated to 120 °C for ~10 min and subsequently allowed to cool to room temperature. Thickness of the Nafion layer was determined to be 1 μm through SEM characterization of a cleaved chip.

### Preparation of ionic transistors with diameter above 200 nm

A second chip with a 40 nm thin layer of silicon nitride was aligned on top of the Nafion-coated chip from the previous step using an optical microscope. In the case of the devices shown in Figs. 4 and 5 the second chip also contained a spin-coated Nafion film. The stack was then bonded together using superglue (Scotch) (ethyl cyanoacrylate) and allowed to dry for 24 h. In the next step, a nanopore was drilled through the full stack using focused ion beam (Tescan, Gaia3). The following conditions were used to drill a 0.25 μm in radius pore: beam current of 0.9 nA, and a depth of 10 μm. The device was subsequently subjected to chemical reaction with (3-aminoproyl) trimethoxysilane, 97%, Sigma Aldrich), which was used as purchased. The devices were immersed for 30 min in a 1% solution of the silanes in ethanol. After rinsing the devices with ethanol, they were placed on a heat plate and heated to 120 °C for 1–3 h.

### Preparation of ionic transistors with diameter below 200 nm

The process started with FIB drilling of single nanopores in two 40 nm thick silicon nitride films; one of the chips contained a prior deposited Au electrode, as described in the section that details preparation of the gate. The single nanopore chips were chemically modified with (3-aminoproyl) trimethoxysilane, as described above. Nafion was subsequently spin-coated on the two chips, which were then bonded together (see the previous section).

### Measurements of ion current

Current-voltage curves were recorded with Keithley 6487 picoammeter/voltage source (Keithley Instruments, Cleveland, OH) using two commercially available Ag/AgCl electrodes (A-M Systems, WA). The voltage was changed between −1 V and +1 V with 0.1 V steps or between −5 V and +5 V with 0.5 V steps. A Keithley 2450 was used to supply voltage or current signal to the gate. Each device was subjected to four voltage scans, which were used to calculate error bars as standard deviation. The first scan was typically unstable thus the averages were performed based on subsequent scans.

### Numerical modeling

Ionic transport in this system was modeled by the coupled Poisson-Nernst-Planck and Stokes-Brinkman equations. The region of the device filled with Nafion was input as a space charge distribution and included in the Poisson equation^32,47^. This set of coupled equations was solved numerically using the finite element software COMSOL Multiphysics. More details about the governing equations and boundary conditions used are provided in Supplementary Notes 1 and 2, Supplementary Table 1, and Supplementary Figs. 5–10, 12.

## Supplementary information

Supplementary Information

## Data Availability

The data that support the findings of this study are located at Dryad repository with the identifier 10.7280/D19T0C.

## Code availability

COMSOL modules associated with the findings of this study are available from the corresponding authors upon request.
