# Supplementary material for: Ionic amplifying circuits inspired by electronics and biology
Source: Nat Commun. 2020 Mar 26;11:1568. doi: 10.1038/s41467-020-15398-3 (PMC7099069; doi:10.1038/s41467-020-15398-3)
Supplement: Supplementary file 1 — Supplementary Information [file 41467_2020_15398_MOESM1_ESM.pdf]

Supplementary Information

Ionic Amplifying Circuits Inspired by Electronics and Biology

Rachel A. Lucas[1], Chih-Yuan Lin,[1] Lane A. Baker,[2] Zuzanna S. Siwy[1,3,4*]

[1]*Department of Physics and Astronomy, 4129 Frederick Reines Hall, University of California, Irvine, CA 92697*

[2]*Department of Chemistry, Indiana University, 800 E. Kirkwood Avenue, Bloomington, IN 47405, United States*

[3]*Department of Chemistry,* [4]*Department of Biomedical Engineering, University of California, Irvine, CA 92697*

[*] Corresponding Author: zsiwy@uci.edu, Tel. 949-824-8290

Supplementary Figures

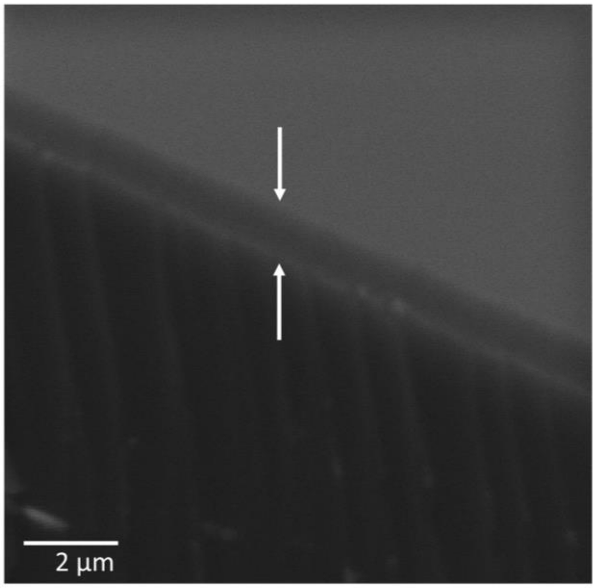

Supplementary Figure 1. **An image of a cleaved silicon nitride chip with Nafion layer on top**. Spin coating conditions were the same as in preparation of the transistor devices. White arrows indicate the Nafion layer. The image was taken with scanning electron microscope (Tescan GAIA3).

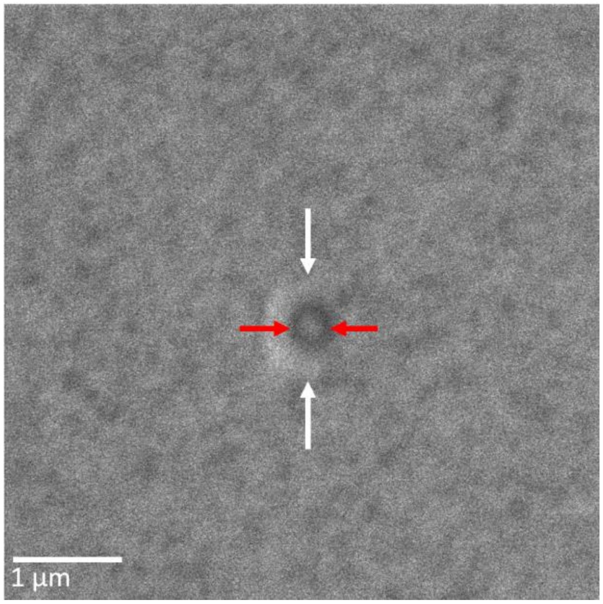

Supplementary Figure 2. **Estimation of a Nafion area damaged by electron beam.** A pore drilled with FIB through a silicon nitride chip covered with Nafion. Conditions of the pore fabrication were similar to the conditions applied in preparation of the npn devices. Red arrows indicate the diameter of the pore. White arrows indicate the diameter of the damaged Nafion surrounding the pore.

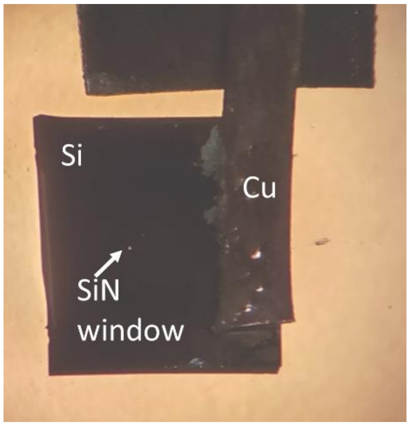

Supplementary Figure 3. **Optical image of an assembled ionic transistor.** The image was taken at 4 times magnification. A white arrow indicates a silicon nitride window where a pore was drilled. The Cu tape provides electrical connection to the Au layer deposited on one of the silicon nitride chips.

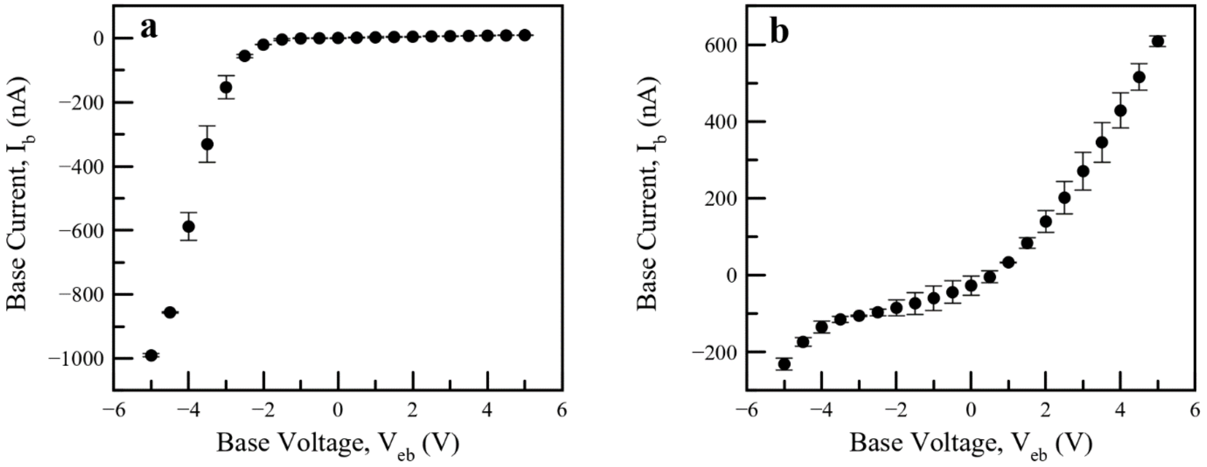

Supplementary Figure 4. **Testing successful chemical functionalization of silicon nitride chips**. Recordings for a transistor with an opening diameter of 600 nm in 10 mM KCl. Current-voltage curves with voltage applied between emitter-base junction with collector electrode disconnected. (a) Measurement with an as prepared device, i.e. pre-silanization; (b) Measurements after silanization, which rendered the silicon nitride surface and pore walls positively charged. Error bars were calculated as standard deviation from an average of at least two independent voltage scans.

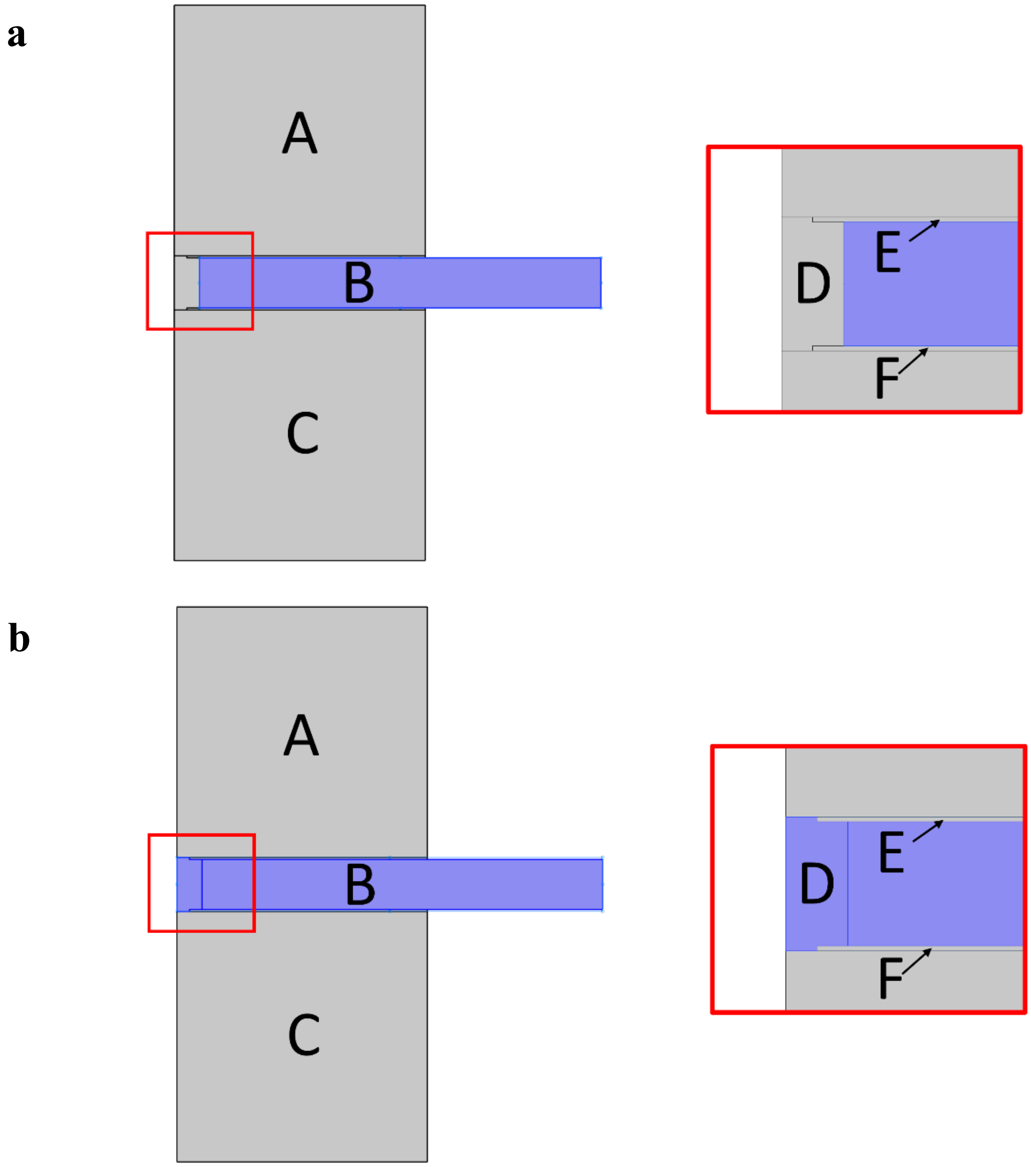

Supplementary Figure 5. **COMSOL simulation model geometry**. A model used for predicting ion transport through a bipolar junction ionic transistor: the total thickness of the devices is 1 µm. Pore diameter is 500 nm for larger devices and 150 nm for smaller devices. Detailed boundary conditions are listed in Supplementary Table 1. (a) Scheme used for large devices (Figure 1c in the main manuscript) and (b) small devices (Figure 1d in the main manuscript) where purple shaded regions are representative of Nafion space charge. In (a) the Nafion region had a diameter twice as large as the silicon nitride pores.

Supplementary Note 1: Modeling System

The main modeling system is illustrated in Supplementary Figure 5, where regions A and C represent identical electrolyte reservoirs. They are connected by a pore, region D, where it was estimated that the Nafion layer would be burned away to give a radius twice as large as that in the silicon nitride region. The nanopore diameter in the silicon nitride layers is 500 nm with a total device length of 1μm. For the larger device area B is the Nafion membrane (Supplementary Figure 5a). For the modeling of the smaller devices region D also included Nafion (Supplementary Figure 5b). Zones E and F are representative of the silicon nitride. The coupled Poisson-Nernst-Planck (PNP) and Stokes-Brinkman (SB) equations are employed to describe this system with the presence of Nafion. Aqueous KCl solution is modeled assuming an incompressible Newtonian fluid so that $\nabla \cdot \mathbf{u} = 0$.

$$-\varepsilon_{\mathrm{f}} \nabla^2 \varphi = \sum_{i=1}^{2} F z_i c_i + h \rho_{\mathrm{PE}} \quad (1)$$

$$\nabla \cdot \mathbf{J}_i = \nabla \cdot \left[\mathbf{u} c_i - D_i \nabla c_i - \frac{z_i F}{RT} D_i c_i \nabla \varphi \right] = 0 \quad (2)$$

$$-\nabla p + \mu \nabla^2 \mathbf{u} - \sum_{i=1}^{2} F z_i c_i \nabla \varphi - h \gamma_{\mathrm{PE}} \mathbf{u} = \mathbf{0} \quad (3)$$

$$\nabla \cdot \mathbf{u} = 0 \quad (4)$$

Supplementary Figure 6 shows geometry used in 3D modeling of a system consisting of two unaligned nanopores, representing the device schematically shown in Figure 1d in the main manuscript. Supplementary Table 1 defines the variable names.

The PNP and SB equations are solved numerically with several assumed boundary conditions. Ionic concentration in the reservoirs essentially reaches the bulk concentration value. The membrane surface is ion-impenetrable and non-slip. Potential biases are applied to the edges of regions C and B while the far-side of region A is grounded so that the ends of C and B correspond to the collector and base respectively while the end of region A corresponds to the emitter. The ionic current is calculated as $\int_{\Pi} \left(\sum_{i=1}^{2} F z_i \mathbf{J}_i \right) \cdot \mathbf{n} \, d\Pi$ where Π represents the end of reservoir C. Region B is modeled with a space charge of $1.5 \cdot 10^5$ C/m^3. This value was chosen based on previous work with polyelectrolytes[1, 2] and reduced by a factor of 100 to achieve convergence of the system. The silicon nitride surfaces of regions E and F are given a surface charge density of +0.04 C/m^2 for the silanized device, a value that is close to the magnitude of surface charge of as prepared silicon nitride nanopores.[3]

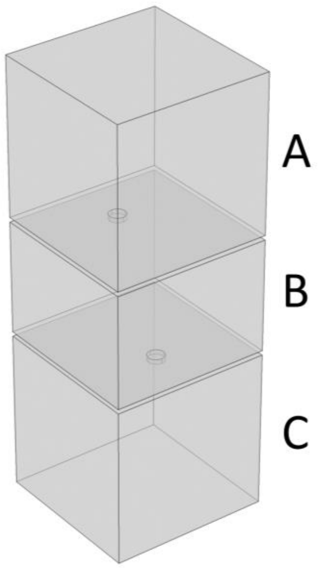

Supplementary Figure 6. **COMSOL simulation model for 3-dimensional calculations**. A model used for predicting ion transport through a bipolar junction ionic transistor: the pores in silicon nitride are 40 nm in length each, and their diameter is 150 nm. Similar boundary conditions are used as for the axisymmetric model in Supplementary Figure 5. Regions A and C are representative of electrolyte reservoirs while region B contains the Nafion membrane as a space charge. In the model shown the pores are offset by 500 nm.

Variable	Description
$\boldsymbol{\varphi}$	Electric potential
$\mathbf{u}$	Fluid velocity
$\mathbf{J}_i$	Flux of ith ionic species
c_i	Concentration of ith ionic species
D_i	Diffusivity of ith ionic species
z_i	Valence of ith ionic species
ε_{f}	Fluid permittivity
F	Faraday constant
R	Gas constant
T	Absolute temperature
p	Hydrodynamic pressure
μ	Dynamic fluid viscosity
ρ_{PE}	Fixed volume charge density of polyelectrolyte layer
h	1 (0) for the region inside (outside) the polyelectrolyte layer
γ_{PE}	Hydrodynamic frictional coefficient of polyelectrolyte layer

Supplementary Table 1. **Definition of all variables used in the modeling**. The variables were used in the numerical model described in the Supplementary Note 1.

Supplementary Note 2: Charge Density Measurements of Silicon Nitride Nanopores

The magnitude of surface charge density was determined for an as prepared nanopore, and a nanopore subjected to symmetric silanization leading to positively charged pore walls and both membrane surfaces; the same chemical modification was performed with chips used to assemble devices shown in Figure 1d. Supplementary Figure 7 shows conductance through an FIB drilled nanopore with an opening diameter of 70 nm before and after silanization; ion current in KCl concentrations between 1 M and 0.1 mM is shown. As reported before, if a pore is charged, the conductance does not follow a linear behavior in the whole range of salt concentrations, but rather at low concentrations, the curve starts to saturate indicating a regime where the pore conductance is governed by surface charge.[3] The experimental data were fit with an analytical formula that relates the pore conductance, G, with the magnitude of surface charge, σ:[4]

$$G = \frac{\pi}{4}\frac{d_{\mathrm{pore}}^{2}}{L_{\mathrm{pore}}}\left((\mu_{\mathrm{K}} + \mu_{\mathrm{Cl}})n_{\mathrm{KCl}}e + \mu_{\mathrm{K}}\frac{4\sigma}{d_{\mathrm{pore}}} + \frac{1}{R_{\mathrm{access}}}\right) \qquad (5)$$

where d_{pore}, and L_{pore}, represent the diameter and length of the pore. n_{KCl} is the number density of potassium ions (equal to the number density of chloride ions) and is determined by the bulk concentration. μ_{K} and μ_{Cl} are mobilities of both ions that we assumed equal to the bulk values. R_{access} is the access resistance that needs to be considered in pores with low aspect ratio.[5]

Fitting the experimental data (Supplementary Figure 7) with eq. 5 showed that the surface charge density before and after silanization is similar and equal to ~|0.02| C/m^2. We confirmed that results of our numerical modeling performed when using 0.02 and 0.04 C/m^2 do not differ by more than 5%.

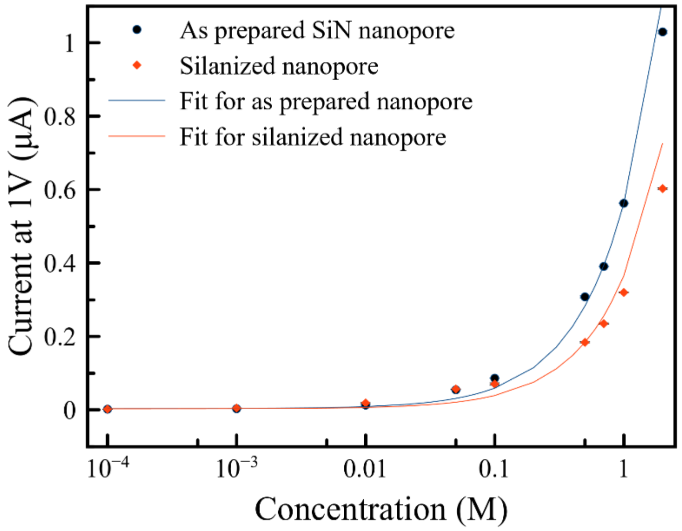

Supplementary Figure 7. **Surface charge density of silicon nitride nanopores**. Experimental data of ion current at 1 V as a function of bulk concentration of KCl at pH 8 are shown as points for an as prepared (black points) and silanized (red points) nanopore. The lines are a fit of the data with eq. (5) for a surface charge density of |0.02| C/m^2.

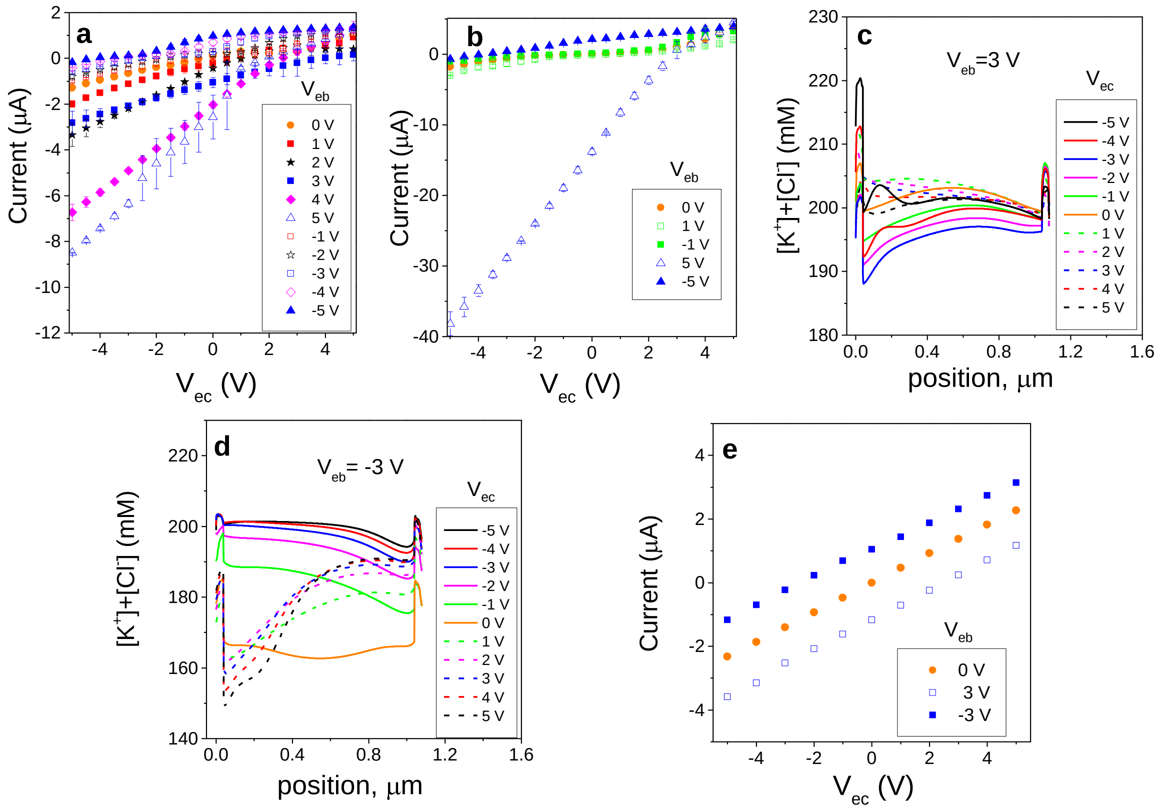

Supplementary Figure 8. **Functioning of an ionic bipolar transistor in 100 mM KCl**. (a) Experimental current-voltage curves with various base voltage inputs for a transistor with an opening diameter of 600 nm in 10 mM KCl; this is the same device as shown in Figure 2a,b however data for more gate voltages, V_{eb}, are included. (b) The same device as in (a) and probed in 100 mM KCl. Error bars in (a) and (b) were calculated as standard deviation from an average of at least two independent voltage scans. (c-e) Results of numerical modeling for a transistor based on 500 nm in diameter pores in 100 mM KCl. These panels should be compared with Figure 3 a-c, which show modeling for the same device in 10 mM KCl.

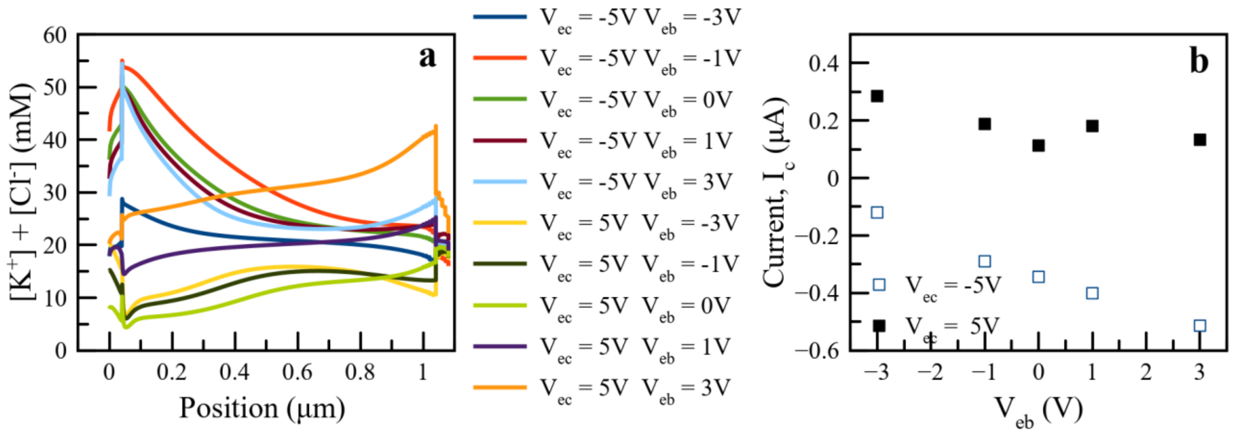

Supplementary Figure 9. **Modeling of ionic concentrations and current in an ionic transistor**. COMSOL modeling of a device consisting of two 40 nm thick silicon nitride films with a 500 nm in diameter pore, and 1 µm thick Nafion layer placed between the chips. The modeling was performed in 10 mM KCl as a bulk solution. (a) Average concentration profile along the pore axis for the silanized device. (b) Collector current as a function of base voltage.

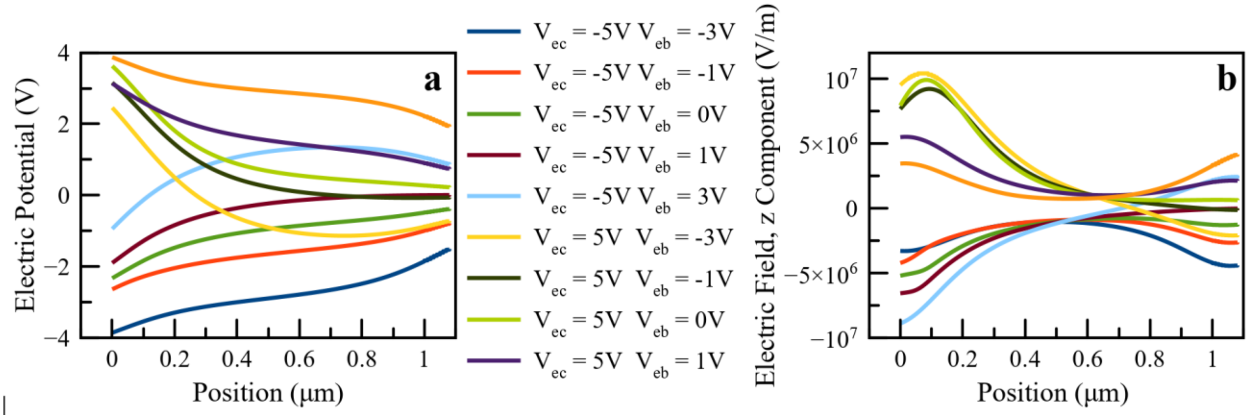

Supplementary Figure 10. **Modeling of electric potential and field in an ionic transistor**. COMSOL modeling of the same system as shown in Supplementary Figure 9. (a) Electric potential along the axis of a silanized device. (b) z-component of electric field along the device axis.

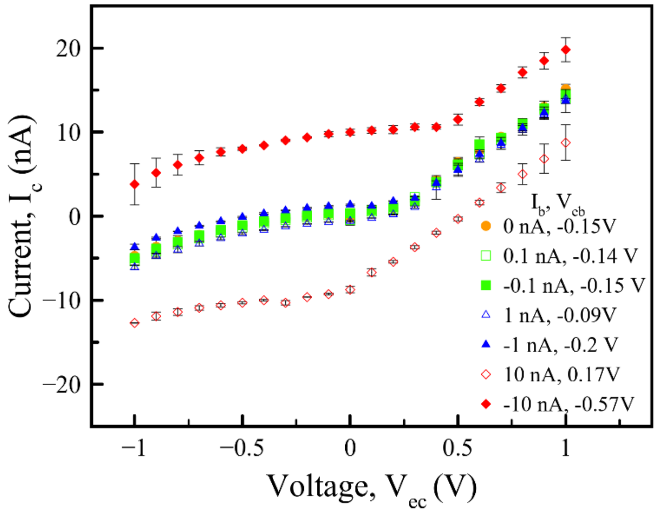

Supplementary Figure 11. Characteristics of an ionic transistor for various base inputs. Experimental recordings for a bipolar junction transistor based on nanopores with an opening diameter of 140 nm; scheme of the device is shown in Figure 1d of the main manuscript. The measurements were performed in 100 mM KCl, pH 8, using gate current, I_b, as the input signal. In order to facilitate comparison of the recordings with data in Figure 2c, corresponding values of gate voltage, V_{eb}, are indicated in the legend as well. Error bars were calculated as standard deviation from an average of values in at least two voltage scans.

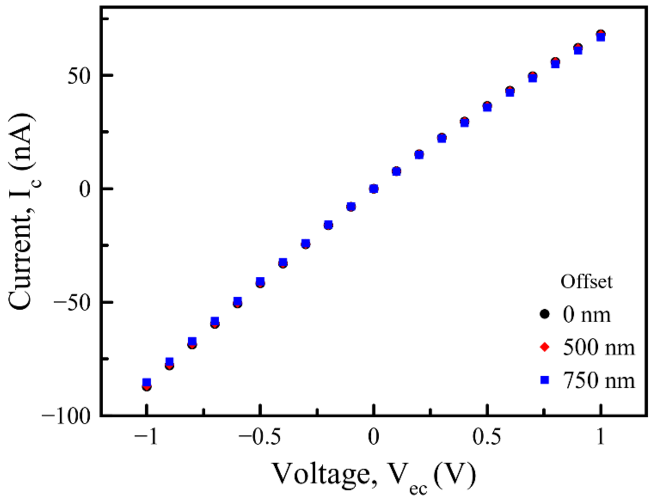

Supplementary Figure 12. **Modeling of ion current in a transistor with two unaligned pores**. Results from the 3-dimensional model shown in Supplementary Figure 6. Collector current-voltage curves shown for pores at various offset distances. 0 nm corresponds to the case when the pores are perfectly aligned.

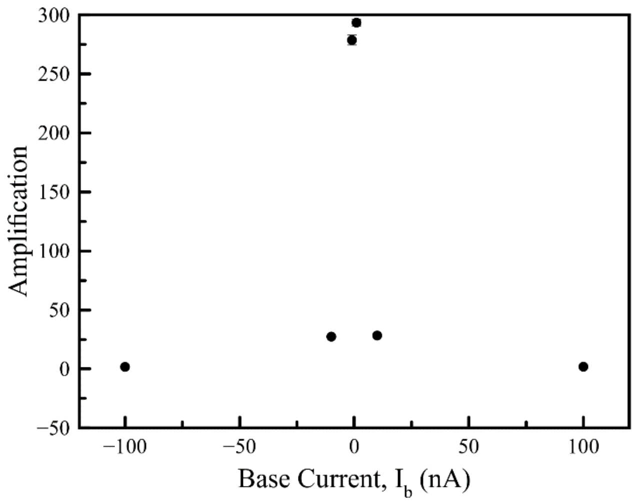

Supplementary Figure 13. **Current gain calculated as a function of base current**. Measurements done for a transistor with an opening diameter of 155 nm in 100 mM KCl. Values calculated for V_{ec} = -3 V at negative base currents, and V_{ec} = 3 V at positive base currents, corresponding to the active modes of the device. Error bars were calculated as standard deviation from an average of values in at least two voltage scans.

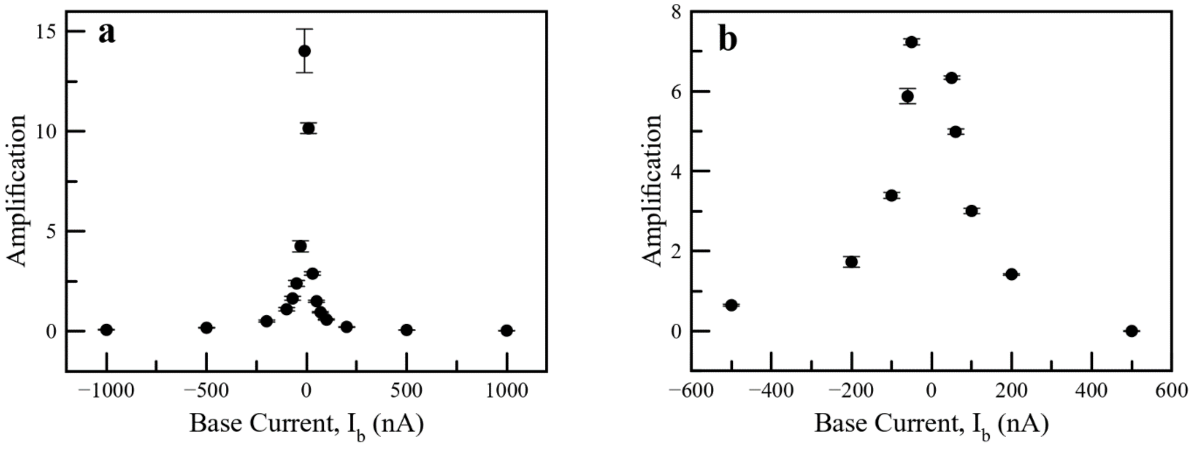

Supplementary Figure 14. **Amplification of individual transistors and the Darlington circuit in 1 mM KCl**. Amplifications of ion current calculated for (a) a bipolar junction containing a 520 nm in diameter pore, and (b) the Darlington setup such that the 520 nm in diameter device provides the input for the 540 nm device, shown in Figure 4 a,b. Note that the Darlington circuit created with the position of the two devices switched, offered gain of ~40 (Figure 5 c). Scheme of individual transistors is shown in Figure 1 c. Error bars were calculated as standard deviation from an average of values in at least two voltage scans.

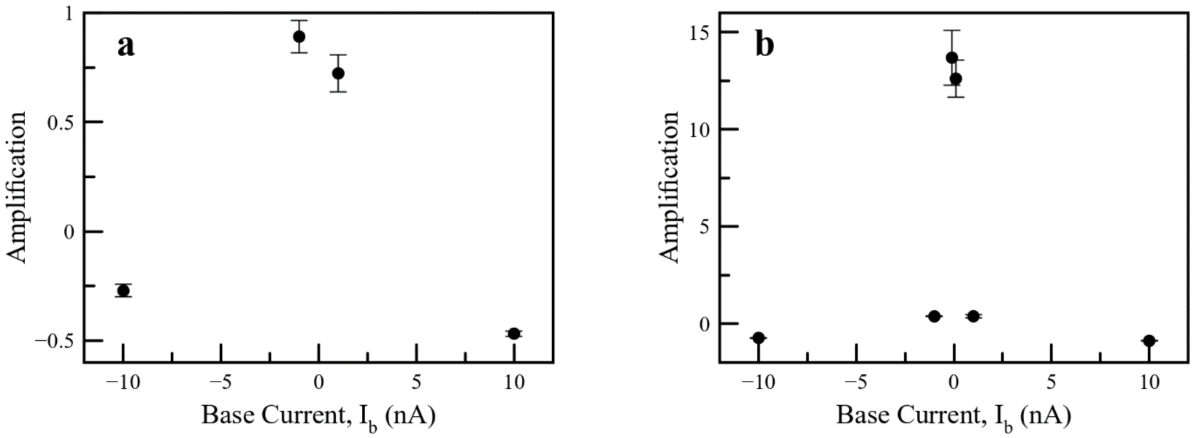

Supplementary Figure 15. **Amplification of individual transistors and the Darlington circuit in 100 mM KCl**. Amplifications of ion current calculated for (a) a bipolar junction containing 150 nm in diameter pores, and (b) the Darlington setup such that a transistor with 150 nm pores provides input for the transistor with 140 nm in diameter pores (Figure 4 c,d). Note that the Darlington circuit created with the position of the two devices switched, offered gain of ~25 (Figure 5 d). Scheme of individual transistors is shown in Figure 1 d. Error bars were calculated as standard deviation from an average of values in at least two voltage scans.

Supplementary References

1. Lin, C.Y., Combs, C., Su, Y.S., Yeh, L.H. & Siwy, Z.S. Rectification of concentration polarization in mesopores leads to high conductance ionic diodes and high performance osmotic power. *J. Am. Chem. Soc.* **141**, 3691-3698 (2019).
2. Barbati, A.C. & Kirby, B.J. Electrokinetic measurements of thin nafion films. *Langmuir* **30**, 1985-1993 (2014).
3. Stein, D., Kruithof, M. & Dekker, C. Surface-charge-governed ion transport in nanofluidic channels. *Phys. Rev. Lett.* **93**, 035901 (2004).
4. Smeets, R.M.M. et al. Salt dependence of ion transport and DNA translocation through solid-state nanopores. *Nano Lett.* **6**, 89-95 (2006).
5. Hall, J.E. Access resistance of a small circular pore. *J. Gen. Physiol.* **66**, 531 (1975).
